# Endophthalmitis in Silicone Oil-Filled Eyes

**DOI:** 10.3390/antibiotics12040736

**Published:** 2023-04-10

**Authors:** Magdalene Yin Lin Ting, Soyang Ella Kim, Rodrigo Anguita

**Affiliations:** 1 Moorfields Eye Hospital NHS Foundation Trust, City Road, London EC1V 2PD, UK; 2 Department of Ophthalmology, Inselspital, University Hospital of Bern, Freiburgstrasse 18, 3010 Bern, Switzerland

**Keywords:** silicone oil, infectious endophthalmitis, pars plana vitrectomy, anti-microbial properties

## Abstract

Incidences of post pars plana vitrectomy (PPV) endophthalmitis vary between 0.02% and 0.13%, and infectious endophthalmitis in silicone oil-filled eyes is even rarer. We performed a literature review to describe the incidence, protective and predisposing factors, causative pathogens, management options, and prognosis of infectious endophthalmitis in silicone oil-filled eyes. Various studies have elucidated different aspects of this condition. Causative pathogens commonly include commensals. Traditional management involves the removal of silicone oil (SO), intravitreal antibiotics and then SO re-injection. Alternatively, injecting intravitreal antibiotics into silicone oil-filled eyes has also been reported. Visual prognoses are uniformly guarded. Due to the uncommon nature of this condition, studies are limited either by their retrospective design or by small sample sizes. However, observational studies, case series, and case reports can play an important role in rare conditions until larger studies are conducted. This comprehensive review aims to summarise the information available in the literature, to act as a good source for ophthalmologists looking for answers on this topic, and to suggest areas for future development.

## 1. Introduction

Infectious endophthalmitis is a severe but rare complication discussed universally on all surgical consent forms in modern ophthalmic surgery, feared by any treating physician while having the potential to be anatomically and visually devastating for the patient. It involves severe inflammation of the internal structures of the eye and ocular tissues, resulting mostly from intraocular propagation of and infection by exogenous microbes, including bacteria, mycetes, or parasites [1,2,3]. These may be introduced into the eye via surgery or via other routes, such as post-trauma or ocular surface infections [3]. Causative agents may be broadly classified into two categories: bacteria and fungi. Multiple factors determine which is the predominant pathogen causing the infection, and these include but are not limited to the source (for example intraocular foreign body or organic matter), route of spread (surgery, trauma, or haematological spread), geographic location, and patient characteristics [4].

Endophthalmitis can be broadly classified into exogenous and endogenous. Post-operative endophthalmitis, a subtype of exogenous endophthalmitis, can be further classified as acute (within six weeks of the surgical date) or chronic [3]. Endogenous endophthalmitis refers to cases in which the infectious pathogen has travelled via the systemic circulatory system to reach the eye [3,5] and is beyond the scope of this paper. Despite ever-growing advances in modern medicine, endophthalmitis remains a major diagnostic and therapeutic challenge within the field of ophthalmic surgery. Due to its sight-threatening nature, it has rightfully been the subject of intensive research, especially in the last few decades.

Several publications have reported local incidences of post pars plana vitrectomy (PPV) infectious endophthalmitis. Over the years, there have been sensible downward trends in the rates, with typical ranges between 0.02 and 0.13% [3,6,7,8]. Amongst the commonly used internal tamponading adjuncts, infectious endophthalmitis in silicone oil (SO)-filled eyes remains very rare. The Pan American Collaborative Retina Study Group observed no cases of endophthalmitis in 7357 SO-filled eyes [9]. 

Only a few studies attempting to elucidate different aspects of this condition have been reported in the literature. Due to the uncommon nature of this condition, these are limited either by their retrospective design or small sample sizes. We performed a literature review to describe the incidence, protective and predisposing factors, clinical features, causative pathogens, management options, and prognosis of infectious endophthalmitis in SO–filled eyes. This comprehensive review aims to summarise the information available in the literature and act as a good source for ophthalmologists looking for answers on this topic. 

## 2. Material/Methods

A literature search was conducted on the PubMed database for publications between the years 1983 and 2022 with the words “endophthalmitis”, “silicone oil”, “surgery”, and “retinal detachment”. “Essential” terms AND, OR were used, yielding a total of 221 articles. Articles in all languages were considered. The remaining abstracts and articles were reviewed by the authors and were included based on their relevance to this review article. In addition, the primary references mentioned in the papers were also reviewed. 

## 3. Silicone Oil—Anti-Microbial Properties

Silicone oils (SOs) are a group of clear, inert, hydrophobic polymers which are chemically composed of repetitive siloxane (Si-O) units [10]. In ophthalmology, they are used as retinal tamponading agents usually reserved for complex retinal detachment repair surgeries, such as those involving proliferative vitreoretinopathy, complex trauma, or detachments related to viral retinitis, as well as in severe cases of infectious endophthalmitis [2]. Among SO’s properties, its antimicrobial activity has been extensively studied. Several in vitro prospective studies have been conducted, whereby SO was inoculated with various bacteria and fungi, and then microorganism growth was charted. These demonstrated that exposure to SO inhibited the growth of microorganisms over 30 days [10,11]. In fact, SO has been reported to hold antimicrobial and fungistatic effects against common causative pathogens of endophthalmitis, including *Staphylococcus aureus*, *Staphylococcus epidermidis*, *Pseudomonas aeruginosa*, *Candida albicans,* and *Aspergillus* spp. [3,10]. Hypothesised mechanisms include nutritional deprivation and toxicity of low-molecular-weight components to microorganism cell membranes [11,12,13,14,15]. Others suggest that SO’s high surface tension and low permeability limit the movement of pathogens, thereby concentrating them close to the ciliary body or blood vessels and allowing defence mechanisms better access [3,16]. Furthermore, SO’s space-occupying action as an endo-tamponade in a long-standing manner could contribute to the washout of pathogens and their toxins by physiological mechanisms, thus preventing substantial damage to retinal tissues [3,17,18]. 

There have also been in vitro studies evaluating SO’s antimicrobial activity against anaerobic pathogens, more specifically *Propionibacterium acnes*, *Peptostreptococcus* spp., *Bacteroides fragilis*, *Fusobacterium* spp., and *Clostridium tertium* [19]. The results from this study show that *Propionibacterium acnes*, which is the most common microorganism causing chronic postoperative endophthalmitis, demonstrates bacterial viability in SO and, hence, resistance to SO’s antimicrobial properties. The paper sets out a few hypotheses for this, including the organism’s biofilm formation capabilities and the production of propionic acid, whose chemical interaction in SO remains unknown.

Ornek et al. suggested in an in vitro study that heavy SO was more effective than conventional SO against common endophthalmitis-causing pathogens [20]. They showed that heavy SO demonstrated a superior antimicrobial effect on all pathogens including *Candida albicans*, whereas conventional SO did not decrease colony numbers of *Candida albicans*. The authors hypothesised that this relates to the hydrophobic and hydrogen bonding interactions of SO. In another study, the chemical composition of SO was suggested to provide antimicrobial properties [21]. The authors proposed this theory since all SOs present an environment with insufficient nutrients for microorganisms, and yet there are differences in antimicrobial properties between different compositions of silicone oil. The exact mechanisms, however, remain poorly understood. A recent systematic review has elucidated the possible lack of SO effectiveness against certain species of fungi, namely *Fusarium* spp., coupled with an absence of proven fungicidal activity [3,22,23].

## 4. Patient Demographics and Risk Factors 

The mean age of patients reported in the literature with endophthalmitis in SO-filled eyes is 44 years (median of 49 years, range of 2–73 years). There is a male to female ratio of 3.5:1 [2,7,8,14,15,24,25,26,27,28]. Of the cases in which laterality was noted, 56% were left eyes and 44% were right eyes. 

The most common indication for initial surgery was rhegmatogenous retinal detachment (78%), associated with proliferative vitreoretinopathy. Other indications included tractional or combined tractional and rhegmatogenous detachment (11%), vitreous haemorrhage secondary to proliferative diabetic retinopathy (6%), and round-hole retinal detachment in chronic Cytomegalovirus retinitis (6%). These patient characteristics are summarised in Table 1.

## 5. Clinical Features

Pain is the most common symptom and was reported in 44% of cases (22% of patients stated they experienced no pain; there were no data or no mention of pain in the remaining 33%); conjunctival hyperaemia was reported in 44% of cases (6% stated they did not experience hyperaemia; there were no data or no mention of hyperaemia in the remaining 50%); anterior chamber cellular activity, keratic precipitates, fibrin, or hypopyon were listed in 61% of cases (there were no data or no mention of these signs in 40%) [2,7,8,14,15,24,25,26,27,28].

An impaired fundal view secondary to the SO becoming opaque was mentioned in 50% of cases. In 17% of cases, there was a view of the fundus, with 6% featuring retinal haemorrhages and 11% mentioning whitish material or exudates on the retinal surface as the main finding. In the remaining 33% of cases, the fundal view was not recorded. 

Other examination findings included lid swelling, ptosis, chemosis, corneal opacity, corneal oedema, and cataracts [2,7,8,14,15,24,25,26,27,28]. 

The onset of endophthalmitis symptoms was within one week post initial surgery in 80% of cases, within one month in 10% of cases, and more than one month in 10% of recorded cases. Visual acuity was reduced to counting fingers in 18% of cases, hand movements in 73% of cases, and perception of light in 9% of recorded cases [2,7,8,14,15,24,25,26,27,28]. These clinical features are summarised in Table 2. 

### Culture Positivity Rates and Organisms

The rates of positive cultures are very low in infectious endophthalmitis in SO-filled eyes [28]. Our literature search demonstrated 12 cases, which are summarised in Table 3. Starting chronologically, a case in 1986 was reported to be caused by *Pseudomonas aeruginosa*. It involved a 5-year-old high myope with a total retinal detachment and a superior giant retinal tear that extended from 270 degrees to 360 degrees by the time of surgery, and hence the decision was made for SO insertion. The patient received surgical management, including the removal of SO, vitreous washout, and intravitreal antibiotics consisting of Gentamicin and Cefazolin, but unfortunately experienced a poor outcome of hypotony and no perception of light [24]. The next case report involved a Cytomegalovirus-related rhegmatogenous retinal detachment in a patient with acquired immunodeficiency syndrome (AIDS), who after a vitrectomy with SO injection, developed infectious endophthalmitis. The aqueous tap was positive for coagulase-negative *Staphylococcus*. The patient received surgical management, with a final visual outcome of 20/100 on the Snellen chart [25]. Another report demonstrates a case of *Pseudomonas aeruginosa* involving a 19-year-old patient with inferior retinal detachment and a 180-degree giant retinal tear who had PPV, an endo-laser treatment, and SO injection. Unfortunately, despite SO removal, a lensectomy, vitreous washout, and intravitreal antibiotics (Vancomycin and Ceftazidime) followed by SO reinjection, their condition continued to worsen. Further intravitreal antibiotics and systemic antibiotics were used and their vision at the end was reported as 20/200, which was maintained at the six-month follow-up [14]. More recently, there was a case series of five patients who developed infectious endophthalmitis after having SO from the same manufacturing batch. The cultures of four of them were positive, growing *Bukholderia cepacie* and *Pseudomonas aeruginosa*. Three of the four cases received topical (Moxifloxacin), systemic (Moxifloxacin orally) and intravitreal antibiotics (Vancomycin 1 mg/0.1 mL and Ceftazidime 2 mg/0.1 mL), and intravitreal steroids (Dexamethasone), as well as delayed surgical interventions due to an initial presumption of sterile inflammation. These cases were all associated with poor anatomical and visual outcomes. The final case was treated with an early surgical intervention (SO removal, multiple irrigation, intravitreal antibiotics and steroids followed by 10% C3F8 tamponade) within two weeks and systemic antibiotics because of the heightened index of suspicion. This patient achieved a final visual acuity of 20/30 [2]. In 2019, a rare case of culture-positive infectious endophthalmitis in SO-filled eyes was reported to involve *Mucormycosis* after PPV with an encircling band. As part of their management, the band was explanted, and intravitreal Vancomycin, Ceftazidime, and Amphotericin B as well as systemic antifungals were administered. The patient declined further surgery and developed phthisis bulbi [15]. A single-arm cohort study in the Middle East highlighted two cases of infectious endophthalmitis in SO-filled eyes, both being young males who had PPV with SO tamponade for retinal detachments. Both cases were managed with a vitreous tap and injection of the antibiotics Vancomycin and Ceftazidime. The cultures grew *Staphylococcus epidermidis*, and their final vision was recorded as 20/40 and 20/100 [8]. In 2021, a Chinese group reported a case involving mixed infections. The 62-year-old female had been treated in an external unit for endophthalmitis secondary to *Staphylococcus epidermidis* cultured from a conjunctival swab, with intravitreal Vancomycin and Ceftazidime. The condition of the eye continued to worsen, and the decision was made to repeat PPV. This showed the lens nucleus in the posterior segment and a subretinal abscess. Silicone oil was exchanged, and the abscess was extracted and cultured, growing *Morganella morganii*. The patient’s final visual acuity was PL [7]. The most recent case report in 2022 was of a healthy patient with culture-positive *Streptococcus pneumoniae* who had initiated standard surgical management. Their final vision, however, was PL, after the patient opted not to have any further interventions despite evidence of a further retinal detachment at the follow-up [28].

## 6. Management

The key points in endophthalmitis management are infection control, inflammation management, and re-infection prevention. Examples of factors requiring thoughtful consideration as they may impact the choice of management strategy include visual potential, the severity of endophthalmitis, microbiology, media clarity, and patient preference [29].

Intravitreal antibiotics remain the mainstay for endophthalmitis management [30,31]. In fact, experimental studies in rabbit eyes as early as the 1940s exist in which intraocular penicillin and sulphonamides were used in the treatment of endophthalmitis [32]. The use of intravitreal antibiotics as a standard is encouraged by the authors of the Endophthalmitis Vitrectomy Study published in 1995, deemed one of the first landmark studies in post-operative cataract surgery bacterial endophthalmitis [33,34]. The most commonly injected antibiotics include Vancomycin 1 mg/0.1 mL (Gram-positive cover), Ceftazidime 2.25 mg/0.1 mL, or Amikacin 0.4 mg/0.1 mL (Gram-negative cover) [3].

Currently, the most commonly chosen management strategy involves re-operation to remove the oil, and the administration of intravitreal antibiotics followed by the re-injection of SO [2,25]. In contrast to such aggressive surgical interventions, there has been a recent report of a successful alternative treatment with intravitreal antibiotics in an outpatient setting. It is thought that the presence of SO complicates management because it precludes vitreous aspirate and the accurate delivery of an appropriate concentration of intravitreal antibiotics, which may lead to severely high concentrations in the retro-silicone space and therefore retinal toxicity [14]. There is currently no consensus on the acceptable dosages of intravitreal antibiotics to be used in SO-filled eyes [28]. A study on retinal toxicity induced by Vancomycin, Ceftazidime, and Ganciclovir in SO-filled rabbit eyes provided evidence that toxicity resulted from half or full dosages, whereas this was not the case for a quarter dosage [35]. Animal studies in macaques showed that intravitreal Vancomycin (1 mg/0.1 mL) and Ceftazidime (2 mg/0.1 mL) had higher peak concentrations in aqueous humour (543.5 μg/mL and 1176.3 μg/mL) and shorter half-lives (6.8 h and 3.1 h) in SO-filled eyes compared with normal eyes (maximum concentration of 151.4 μg/mL and 64.6 μg/mL; half-life of 29.4 h and 20.4 h) [36]. Their pharmacokinetics simulation supported this, showing that in normal eyes, the maximum drug concentration in aqueous humour peaked at 322 μg/mL with a half-life of 12.8 h, whereas the maximum drug concentration was 1250 μg/mL with a half-life of 3.3 h in SO-filled eyes. Furthermore, they reported no changes in ERG patterns after intravitreal antibiotic injections in SO-filled eyes; hence, they did not report retinotoxic effects. It is worth noting, however, that these results may not be applicable to human eyes [29].

Steinmetz et al. presented two cases which showed the successful resolution of endophthalmitis with a single injection of either half-dose (0.5 mg Vancomycin and 1.13 mg Ceftazidime) or full-dose (1.0 mg Vancomycin and 2.25 mg Ceftazidime) intravitreal antibiotics alone into the SO-filled vitreous cavity in the office, with no clinical evidence of retinal toxicity and resultant visual acuities of 20/80 and 20/400 [27]. Such a minimally invasive approach to endophthalmitis in SO-filled eyes management could be advantageous, especially if surgical interventions cannot take place immediately. Nonetheless, it would be prudent to exercise caution in generalising this therapeutic alternative drawn from this single report. However, there is also a limit on the anti-microbial activity of SO, and a case series has demonstrated how the prompt removal of SO with the lavage of the vitreous cavity and repeat tamponade when there is suspicion of *Bukholderia cepacie* can have good results [2]. On the other hand, patient refusal for surgical intervention in a case of *Mucorales* spp. endophthalmitis was shown to lead to phthisis bulbi, though this microorganism is known to be very virulent so this could well have been the main cause of the poor outcome [15]. It has also been suggested in the literature that should there be suspicion of mixed infections, an early re-operation with a vitrectomy needs to be considered [7].

An Indian retrospective review of over 100,000 vitrectomy cases showed that a substantial proportion of culture-negative cases could be effectively managed with intravitreal antibiotic injections with both better anatomic and better visual outcomes [37]. However, endophthalmitis in SO-filled eyes is a different entity, and further studies are needed to provide guidance as to whether we may extrapolate these data directly.

Due to the lack of evidence, clinical judgement is advised when choosing between urgent surgical intervention compared to immediate intravitreal antibiotics for endophthalmitis in SO-filled eyes.

The use of systemic antibiotic therapy as an adjunct in the treatment of bacterial endophthalmitis is controversial. For the successful elimination of the infection, the antibiotics administered must be able to reach intraocular tissues [38]. Multiple physiological protective barriers significantly obstruct the penetration of topical and systemic antibiotics into the intraocular space. Hence, satisfactory drug concentrations can most usually be achieved via the intravitreal route, which provides direct access to the vitreous cavity and bypasses the blood–retinal barrier [38,39,40]. To list specific examples, topical medications are prone to dilution by the tear film and removal by lacrimal flow [41] as well as the systemic absorption and removal by conjunctival capillaries and nasolacrimal mucosal surfaces [42], while the tight junctions on the corneal epithelium prevent paracellular drug penetration especially for ionic medications [43]. As for systemically administered medications, they may gain access to the choroidal extravascular space but thereafter, their distribution remains impeded by the retinal pigment epithelium (RPE) and the retinal endothelium [44]. Without adequate antimicrobial concentrations, irreversible ocular tissue destruction and injury may ensue [38,39]. A caveat to this is the fact that there are insufficient studies evaluating concentrations achievable in inflamed eyes in which there is breakdown of the blood–retinal barrier.

There remains a poor evidence base to support the use of specific antibiotic regimens. Although there is a lack of empirical evidence on clinical efficacy and official published univocal guidance, the prescription of systemic therapy remains relatively common given the severity of the condition, and such prescribing behaviour may result in the inappropriate treatment of endophthalmitis whilst contributing to poor antibiotic stewardship [45]. An EVS study in 1995 concluded that systemic ceftazidime (dosage of 2 g every 8 h) and amikacin (dosage of 7.5 mg/kg initially followed by 6 mg/kg every 12 h) did not positively influence final visual outcomes. Moreover, the study also set out a hypothesis that the omission of systemic therapy could decrease risks of toxicity and unnecessary financial costs [33,34]. Examples of well-documented systemic antibiotic regimens that appear to achieve intravitreal therapeutic levels include fourth-generation fluoroquinolones, meropenem, and linezolid [38,39,46,47,48]. We anticipate that future advances in ocular drug delivery system research will lead to improved drug penetration, bioavailability, and efficacy, resulting in the furthering of patient safety and options of less invasive administration. Until then, intravitreal antibiotics remain the mainstay of endophthalmitis treatment, as discussed.

Should the eye be deemed unsalvageable or prove to fail to recover from attempted interventions or the infection is at a high risk of extraocular spread, enucleation is typically indicated as a last resort. Reports have quoted endophthalmitis as a common (9.1 to 27.3%) indication for enucleation, behind other causes such as trauma, tumours, glaucoma, and phthisis bulbi [49]. A retrospective study of 210 cases of endophthalmitis found that endogenous endophthalmitis was a risk factor strongly associated with evisceration or enucleation, whilst post-operative endophthalmitis cases were less likely to warrant either of these [49]. Moreover, the delayed diagnosis and management of endophthalmitis have also been associated with the need for enucleation or evisceration [49,50].

## 7. Prognosis

Due to the uncommon nature of endophthalmitis in SO-filled eyes, it is difficult to draw definitive conclusions about its prognosis. Based on the case reports discussed above, the tendency is towards a very guarded visual prognosis, with the majority of patients in these cases having perception of light (PL) vision or worse. A retrospective, multi-centre, noncomparative clinical case series of five large tertiary referral retinal practices in the United States over a period of four years reported the visual outcomes of acute endophthalmitis in 70 patients who underwent therapeutic PPV [51]. Fifteen of the seventy eyes underwent silicone oil injection during PPV. At the last follow-up, other than one patient who retained a final visual acuity of 20/40 with an attached retina, all other cases resulted in very guarded visual acuity ranging from counting fingers to no perception of light (NPL) [29,51].

As for any type of infection, one’s prognosis is generally dependent on organism virulence and the spectrum of antibiotic sensitivity. Other factors that influence one’s prognosis include associated retinal detachment, the presence of advanced proliferative vitreoretinopathy, hypotony, phthisis bulbi, and corneal opacification [29]. Visual outcomes are uniformly guarded, with only an estimated 28% of patients in these cases regaining a visual acuity of 20/50 or better on the Snellen chart [1,14].

## 8. Conclusions

We highlight and comprehensively summarise the information currently available in the wider literature on the topic of infectious endophthalmitis in SO-filled eyes. Traditional management involves re-operation to remove the SO, the administration of intravitreal antibiotics, and re-injection of SO. On the other hand, there has been a recent case report of a successful alternative treatment with intravitreal antibiotics in an outpatient setting which necessitates a cautious interpretation. Evisceration or enucleation remain last resorts, while the systemic administration of antibiotic therapy remains controversial with poor evidence. Therapies used in the treatment of endophthalmitis should be modified accordingly and in a timely manner, depending on culture positivity and sensitivity results, as well as clinical response [52]. Most of the patients in the cases presented in this paper show guarded visual prognoses. Infectious endophthalmitis in SO-filled eyes remains a very rare occurrence. As such, there remains a paucity of publications and studies, which are limited either by their retrospective design or small sample sizes. However, observational studies, case series, and case reports play an important role in rare conditions until larger studies can be conducted.

## Figures and Tables

**Table 1:** Summary of patient characteristics from previous reports of endophthalmitis in silicone oil-filled eyes.

	Reference	Patient	Age (Years)	Sex	Eye	PMH	Initial Diagnosis
1.	Chong, 1986 [24]	A	5	Female	L	-	Total RD, GRT
2.	Zimmer-Galler, 1997 [25]	B	49	Male	L	AIDS, CMV retinitis	Round hole RD
3.	Oshima, 2010 [26]	C	64	Male	L	-	RRD
		D	53	Male	L	-	VH, PDR
4.	Goel, 2015 [14]	E	19	Male	R	-	RD, GRT
5.	Okonkwo, 2018 [2]	F	60	Female	R	-	Chronic RD, PVR
		G	34	Female	R	-	Chronic RD
		H	34	Male	R	-	Chronic RD, PVR
		I	43	Male	L	-	RD, PVR
		J	63	Male	L	-	Chronic RD
6.	Steinmetz, 2018 [27]	K	56	Male	?	-	RD
		L	61	Male	?	-	RD, PVR
7.	Dogra, 2019 [15]	M	?	Male	R	-	Total RD
8.	AlBloushi, 2021 [8]	N	38	Male	L	Bronchial asthma	RRD
		O	29	Male	R	Diabetes mellitus	CTRRD
		P	2	Male	R	Knobloch syndrome	RRD
9.	Xiao, 2021 [7]	Q	62	Female	L	Hypertension	VH, BRVO, TRD
10.	Al Taisan, 2022 [28]	R	73	Male	L	Alzheimer disease	Chronic RRD

PMH: past medical history; R: right; L: left; RD: retinal detachment; GRT: giant retinal tear; RRD: rhegmatogenous retinal detachment; VH: vitreous haemorrhage; PDR: proliferative diabetic retinopathy; PVR: proliferative vitreoretinopathy; CTRRD: combined tractional and rhegmatogenous retinal detachment.

**Table 2:** Summary of clinical features from previous reports of endophthalmitis in silicone oil-filled eyes.

	Reference	Patient	VA at Diagnosis	Symptoms	Signs	Impaired Fundal View	Final VA
1.	Chong, 1986 [24]	A	-	Pain	Conjunctival injection, AC filled with silicone oil, white material on retinal surface	No	NPL
2.	Zimmer-Galler, 1997 [25]	B	HM	Pain	Increased IOP, keratic precipitates, AC cells, hypopyon, cataract, no fundal view	Yes	20/100
3.	Oshima, 2010 [26]	C	-	Pain	Fibrin, hypopyon, retinal haemorrhages	-	20/200
		D	HM	Blurred vision	Fibrin, hypopyon	-	20/30
4.	Goel, 2015 [14]	E	CF	No pain, no periorbital or palpebral swelling, no conjunctival injection	Hypopyon, hazy media	Yes	20/200
5.	Okonkwo, 2018 [2]	F	HM	Ptosis, erythema, no pain	Erythema, keratic precipitates, opaque silicone oil	Yes	PL
		G	HM	Pain, conjunctival hyperaemia, ptosis	Ptosis, keratic precipitates, hazy fundal view	Yes	HM
		H	PL	Watering. conjunctival hyperaemia, lid oedema, no pain	Hyperaemia, chemosis, corneal opacity, keratic precipitates, hypopyon, cataract, posterior synechiae, corneal stromal abscess	Yes	NPL
		I	HM	Ptosis, lid swelling, hyperaemia, no pain	Chemosis, corneal ulcer, AC flare, hypopyon	Yes	HM
		J	HM	Pain, redness	Hyperaemia, keratic precipitates, opaque silicone oil	Yes	20/30
6.	Steinmetz, 2018 [27]	K	HM	Pain, reduced vision	AC cells, flare, fibrin, hypopyon, no fundal view	Yes	20/80
		L	HM	Pain, reduced vision	Hypopyon	-	20/400
7.	Dogra, 2019 [15]	M	-	Reduced vision	Hyperaemia, SO globules in AC, subconjunctival exudates, retinal exudates	No	NPL
8.	AlBloushi, 2021 [8]	N	HM	-	-	-	-
		O	HM	-	-	-	-
		P	-	-	-	-	-
9.	Xiao, 2021 [7]	Q	-	Pain, redness, reduced vision,	Lid swelling, conjunctival chemosis, corneal opacity, keratic precipitates, hypopyon, fixed pupil, posterior synechiae, cataracts, raised IOP, no fundal view	Yes	-
10.	Al Taisan, 2022 [28]	R	HM	Redness, swelling	Lid swelling, conjunctival chemosis, corneal oedema, 4 + AC cells, hypopyon, fibrin, poor fundal view	Yes	PL

VA: visual acuity; R: right; L: left; IOP: intraocular pressure; CF: counting fingers; HM: hand movements; PL: perception of light; NPL: no perception of light; AC: anterior chamber.

**Table 3:** Summary of culture positivity from previous reports of endophthalmitis in silicone oil-filled eyes.

Reference	Patient	Initial Diagnosis	Cultured Organism	Initial Management
Chong, 1986 [24]	A	Total RD, GRT	Exudate on retinal surface: *Pseudomonas aeruginosa*	Surgical exchange of SO, vitreous washout, IVT Gentamicin and Cefazolin
Zimmer-Galler, 1997 [25]	B	Round hole RD in CMV retinitis	Aqueous: Coagulase-negative *Staphylococcus*	Surgical exchange of SO, IVT Vancomycin and Cefazolin
Goel, 2015 [14]	E	RD, GRT	Exudate on retinal surface: *Pseudomonas aeruginosa*	Surgical exchange of SO, IVT Vancomycin and Ceftazidime
Okonkwo, 2018 [2]	G	Chronic RD	SO, vitreous: *Pseudomonas* spp.	Topical, systemic, IVT Vancomycin, Ceftazidime and Dexamethasone
	H	Chronic RD, PVR	SO, vitreous: *Burkholderia cepacia*	Topical, systemic, IVT Vancomycin, Ceftazidime and Dexamethasone
	I	RD, PVR	SO, vitreous: *Burkholderia cepacia*	Topical, systemic, IVT Vancomycin, Ceftazidime and Dexamethasone
	J	Chronic RD	SO, vitreous: *Pseudomonas aeruginosa*	Surgical SO removal, IVT Vancomycin, Ceftazidime and Dexamethasone, C3F8 tamponade
Dogra, 2019 [15]	M	Chronic total RRD	Subconjunctival exudates: *Mucormycosis*	Encircling band explanted, IVT Vancomycin, Ceftazidime and Amphotericin B, systemic antifungals
AlBloushi, 2021 [8]	N	RRD	Vitreous: *Staphylococcus epidermidis*	IVT Vancomycin and Ceftazidime
	O	CTRRD	Aqueous: *Staphylococcus epidermidis*	IVT Vancomycin and Ceftazidime
Xiao, 2021 [7]	Q	VH, BRVO, TRD	Conjunctival swab: *Staphylococcus epidermidis*; Subretinal abscess: *Morganella morganii*	IVT Vancomycin and Ceftazidime; Surgical exchange of SO, lensectomy, removal of subretinal abscess
Al Taisan, 2022 [28]	R	Chronic RRD	Aqueous, SO and vitreous: *Streptococcus pneumoniae*	Surgical exchange of SO, IVT Vancomycin and Ceftazidime

## Data Availability

Not applicable.
